# Development and preliminary validation of an *omp25*-based RPA-CRISPR/Cas12a visual assay for rapid screening of brucellosis-associated *Brucella* DNA

**DOI:** 10.3389/fmicb.2026.1986052

**Published:** 2026-09-29

**Authors:** Yu Zhang, Shuairan Zhang, Cai Yin, Minjing Jin, Xiaona Zang, Yawen Wu, Taotao Bai, Long Ma, Xiaoliang Wang, Yong Shi, Zhixin Li

**Affiliations:** 1 College of Animal Science and Technology, Ningxia University, Yinchuan, China; 2 Ningxia Hui Autonomous Region Animal Disease Prevention and Control Center, Yinchuan, China; 3 College of Life Science and Technology, Ningxia Vocational and Technical University, Yinchuan, China

**Keywords:** bovine nasal swab, Brucella, CRISPR-Cas12a, molecular screening, *omp25*, recombinase polymerase amplification, visual detection

## Abstract

Brucellosis remains an important zoonotic disease for livestock production and public health, creating a need for rapid molecular screening methods with limited equipment requirements. We developed a visual recombinase polymerase amplification (RPA)-CRISPR/Cas12a assay targeting a 642-bp region of the *Brucella omp25* gene. Comparative sequence analysis was used to assess target conservation, after which four RPA primer pairs and two CRISPR RNAs (crRNAs) were experimentally screened. RPA3-crRNA1 produced the strongest fluorescence response and was used for assay optimization. The final Cas12a reaction contained 250 nM Cas12a, 200 nM crRNA, and 200 nM single-stranded DNA fluorescent reporter. RPA amplification and Cas12a detection required 20 min each, and positive reactions produced visible yellow-green fluorescence under 470-nm blue light. No cross-reactivity was observed with the non-target pathogens included in the experimental panel. In silico comparisons with selected phylogenetically related taxa identified sequence differences within the final RPA3 primer-binding sites and crRNA1 recognition region, supporting a low predicted risk of sequence-based cross-reactivity for the taxa examined. Using purified plasmid DNA, the lowest DNA concentration detected under the experimental conditions tested was 2.72 copies/μL of input template, corresponding to an estimated 13.6 copies per RPA reaction. Among 96 bovine nasal swab samples collected during routine surveillance, agreement analysis against the qPCR comparator showed concordant results for 93 of 96 samples, with positive percent agreement (PPA), negative percent agreement (NPA), and overall percent agreement (OPA) of 100.00, 93.75, and 96.88%, respectively; three qPCR-negative samples showed weak RPA-CRISPR/Cas12a fluorescence and could not be independently resolved. Because the target region is conserved in the vaccine strains A19, S19, and RB51, the assay is intended for preliminary screening of brucellosis-associated *Brucella* DNA rather than species identification or differentiation of vaccine-derived and wild-type DNA. Broader validation with additional specimen types and specificity panels is required before routine diagnostic application.

## 1 Introduction

Brucellosis is an important zoonotic disease caused by bacteria of the genus *Brucella*. In livestock, including cattle, sheep, and pigs, infection can result in abortion, infertility, orchitis, retained placenta, and decreased milk production, thereby causing substantial losses to animal production and posing a continuing public-health risk (Rehman et al., 2025). Human infection is mainly associated with direct contact with infected animals, aborted fetuses, placentas, and secretions, as well as consumption of unpasteurized dairy products (Dadar et al., 2019). Common clinical manifestations in humans include fever, sweating, fatigue, and arthralgia, and the disease may become chronic or recurrent (Freire et al., 2024; Zheng et al., 2018).

Current diagnostic methods for brucellosis mainly include bacteriological isolation, serological assays, and molecular detection. Bacterial isolation is highly specific and is widely regarded as a confirmatory diagnostic method; however, it is time-consuming, poses biosafety risks, and generally requires specialized laboratory facilities (Hassan et al., 2022). Serological assays, such as the Rose Bengal plate test, standard tube agglutination test, and enzyme-linked immunosorbent assay, are widely used for herd-level surveillance, although their performance may be influenced by infection stage, vaccination status, sample quality, and cross-reactive antibodies (Khairullah et al., 2024; Khoshnood et al., 2022). PCR and real-time PCR provide sensitive molecular detection of *Brucella* DNA and have been applied to a range of animal specimens (Mabe et al., 2022; Kaynak-Onurdag et al., 2016), but they generally depend on thermal cycling and laboratory-based fluorescence or electrophoretic equipment. Rapid molecular methods with reduced equipment requirements may therefore be useful for preliminary screening in veterinary and resource-limited settings.

Recombinase polymerase amplification (RPA) enables rapid nucleic-acid amplification under isothermal conditions and has the advantages of short reaction time and low equipment requirements (Srivastava and Prasad, 2023). The CRISPR/Cas12a system provides an additional sequence-dependent recognition step: after crRNA-guided recognition of a target containing a compatible protospacer adjacent motif (PAM), activated Cas12a exhibits collateral cleavage activity toward single-stranded DNA reporters (Paul and Montoya, 2020; Manghwar et al., 2019; Li et al., 2018; Swarts and Jinek, 2019). Integration of RPA or related isothermal amplification with CRISPR-based detection has been increasingly explored for rapid pathogen testing (Zhang K. et al., 2023; Ji et al., 2025; Zhang et al., 2022), including applications to African swine fever virus, severe acute respiratory syndrome coronavirus 2, and porcine circovirus (Gao et al., 2025; Xiong et al., 2020; Wang et al., 2024). More recently, recombinase-based amplification coupled with CRISPR/Cas12a has also been applied to the rapid detection of *Mycoplasma* species. An RPA-CRISPR/Cas12a assay has been reported for *Mycoplasma pneumoniae*, using fluorescence and lateral-flow readouts (Li et al., 2025), while a field-deployable RAA-CRISPR/Cas12a platform has been developed for the veterinary pathogen *Mycoplasma gallisepticum* in poultry (Hu et al., 2026). These studies further demonstrate the applicability of recombinase-based isothermal amplification combined with Cas12a detection to rapid molecular screening of both human and veterinary pathogens.

Several conserved genomic targets have been used for molecular detection of *Brucella*. Recent CRISPR-assisted assays have expanded available strategies for rapid *Brucella* detection using different amplification formats and molecular targets (Mao et al., 2025; Shen et al., 2025; Rasool et al., 2026; Deng et al., 2025; Dang et al., 2023). Among established targets, *bcsp31* and IS711 are widely used, while the copy number and genomic distribution of IS711 vary among *Brucella* species and lineages (Becker and Tuon, 2021; Aljanazreh et al., 2023). The *omp25* gene encodes a 25-kDa outer membrane protein and has been investigated in molecular characterization as well as rapid amplification-based detection (Zhang T. et al., 2023; Song et al., 2025). The selected 642-bp *omp25* region was considered technically suitable for the present platform because it showed high conservation among representative classical *Brucella* sequences and contained multiple candidate RPA primer-binding regions together with Cas12a-compatible crRNA target sites, allowing systematic experimental screening of primer-crRNA combinations within a defined protein-coding locus. Accordingly, the rationale for selecting *omp25* was to evaluate a conserved alternative target that was compatible with the RPA-CRISPR/Cas12a workflow.

In the present study, conservation of the *omp25* target region was first evaluated among representative classical *Brucella* strains, followed by experimental screening of RPA primer-crRNA combinations. An *omp25*-based RPA-CRISPR/Cas12a visual assay was then established and optimized, and its analytical performance was assessed together with a preliminary agreement evaluation against qPCR using bovine nasal swab samples collected during routine surveillance. Because the assay recognition region is conserved in the vaccine strains A19, S19, and RB51, the method was evaluated as a screening assay for brucellosis-associated *Brucella* DNA rather than for species identification or differentiation between vaccine-derived and wild-type DNA.

## 2 Materials and methods

### 2.1 Reagents and instruments

A universal DNA purification and recovery kit was purchased from Tiangen Biotech Co., Ltd. (Beijing, China). RPA amplification was performed using the RPA 4.0/RT-RPA 4.0 Basic Nucleic Acid Amplification Reagent (Suzhou Keer Life Technology Co., Ltd., Suzhou, China). LbCas12a (Cpf1) Nuclease (catalog no. 32108) and HOLMES-Fluo ssDNA Reporter 1 (FAM) (catalog no. 31101) were purchased from ToloBio (Shanghai, China). A commercial *Brucella* genus real-time PCR detection kit (probe-based method) was purchased from Qingdao Lijian Biotechnology Co., Ltd. (Qingdao, China).

The main instruments used in this study included a high-speed centrifuge and gel imaging system (Bio-Rad Laboratories, USA), a real-time fluorescence PCR system (QX-OPTi96, Sichuan Jielaimei Technology Co., Ltd., China), an electrophoresis apparatus (Beijing Liuyi Biotechnology Co., Ltd., Beijing, China), an automated nucleic acid extraction system (NP968-S, Xi’an Tianlong Science and Technology Co., Ltd., Xi’an, China), and a blue-light illuminator (QW-GC470, Hangzhou Qiwei Instrument Co., Ltd., Hangzhou, China) with an excitation wavelength of 470 nm.

### 2.2 Construction of the positive standard plasmid

The *omp25* sequence used in this study was retrieved from chromosome I of *Brucella abortus* strain 2308 in GenBank (accession no. NC_007618.1). The *omp25* region corresponds to nucleotide positions 706,319–706,960 and is 642 bp in length. The sequence was analyzed using NCBI BLAST, and the 642-bp fragment encompassing the assay target region was selected for construction of the positive standard. The fragment was chemically synthesized by Tsingke Biotechnology Co., Ltd. (Beijing, China) and cloned into the pUC19 vector. The final recombinant plasmid was 3,352 bp in length, comprising the 2,686-bp pUC19 backbone and the 642-bp *omp25* insert, together with an additional 24 bp associated with the cloning construct. The recombinant plasmid was used as the positive analytical standard for assay establishment, optimization, and analytical sensitivity evaluation.

After plasmid extraction, the DNA concentration was measured as 100 ng/μL. Plasmid copy number was calculated as copies/μL = (C × 6.02 × 10^23)/(L × 660 × 10^9), where C is the plasmid concentration in ng/μL and L is the total recombinant plasmid length in bp. Based on a concentration of 100 ng/μL and a total length of 3,352 bp, the initial pUC19-*omp25* plasmid concentration was calculated to be approximately 2.72 × 10^10 copies/μL.

### 2.3 Conservation analysis of the *omp25* target region

To assess sequence conservation and the predicted coverage of the selected *omp25* target, the 642-bp region from *Brucella abortus* strain 2308 (NC_007618.1:706319–706,960) was used as the reference sequence. Homologous sequences from representative *Brucella* strains and species, including *B. abortus* 544 (NZ_KB850274.1), *B. abortus* S19 (NC_010742.1), *B. abortus* A19 (NZ_CP030751.1), *B. abortus* RB51 (NZ_CP024670.2), *B. melitensis* 16M (NC_003317.1), *B. suis* 1330 (NC_004310.3), *B. canis* ATCC 23365 (NC_010103.1), *B. ceti* DDEO02 (NZ_CP127845.1), and *B. ovis* ATCC 25840 (NC_009505.1), were retrieved from GenBank and aligned using SnapGene. Sequence comparison was performed at two levels. First, the full 642-bp target region was examined to visualize overall conservation and the relative positions of the candidate RPA primers and crRNAs (Figure 1A; Supplementary Figure S1). Second, a focused local comparison was performed for the final RPA3-crRNA1 sites and nearby candidate detection-related regions, including the RPA3 forward- and reverse-primer binding sites, the crRNA1 recognition region, the crRNA2/RPA-F2 candidate region, and the RPA-R2/RPA-R3 region (Figure 1B). The analysis was used to evaluate sequence conservation and predicted compatibility of the assay target sites among the representative *Brucella* sequences examined.

**Figure 1:**
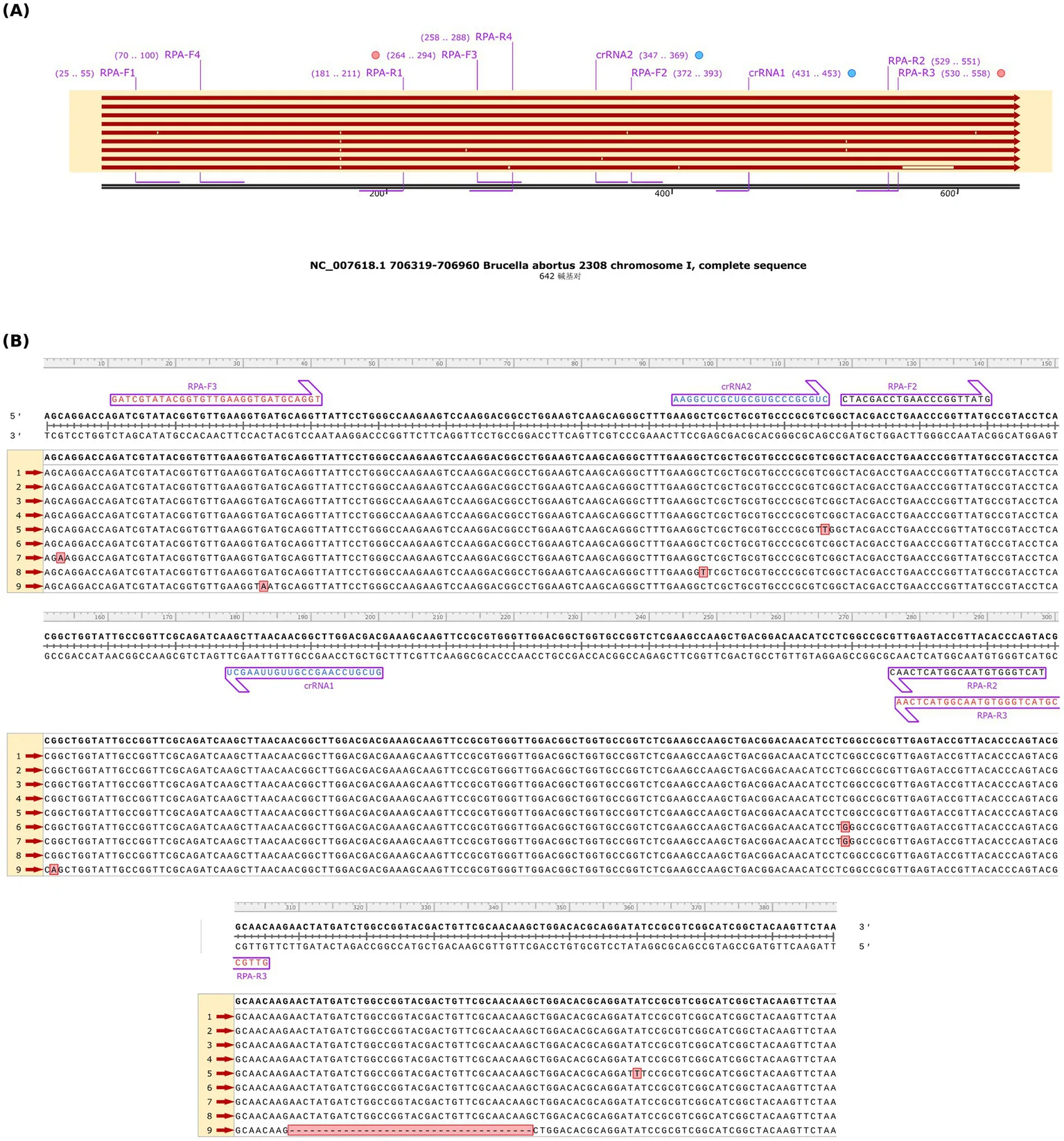
Overview of the 642-bp *omp25* target region and local sequence comparison of candidate and final detection-related sites. **(A)** Schematic overview of the 642-bp *omp25* target region from *Brucella abortus* strain 2308 (NC_007618.1:706319–706,960), showing the locations of the four candidate RPA primer pairs (RPA1–RPA4) and two candidate crRNAs (crRNA1 and crRNA2). The experimentally selected RPA3-crRNA1 combination is indicated. **(B)** Local sequence comparison of the final RPA3-crRNA1 sites and nearby candidate detection-related regions among representative *Brucella* sequences. The RPA3 forward-primer binding site, crRNA2/RPA-F2 candidate region, crRNA1 recognition region, RPA-R2/RPA-R3 region, and the downstream sequence region containing the representative gap in *B. ovis* are shown. Nucleotide mismatches relative to the *B. abortus* 2308 reference sequence are highlighted, and gaps are indicated by dashes. Rows 1–9 correspond to the representative *Brucella* sequences included in the alignment. The complete 642-bp alignment is provided in Supplementary Figure S1.

### 2.4 Design and synthesis of RPA primers and crRNAs

RPA primer pairs were designed based on the *omp25* target sequence of *Brucella abortus* strain 2308 (NC_007618.1:706319–706,960) and synthesized by Tsingke Biotechnology Co., Ltd. (Beijing, China). Four candidate RPA primer sets (RPA1–RPA4) were designed and subjected to experimental screening. The final primer set was selected experimentally based on amplification performance and compatibility within the coupled RPA-CRISPR/Cas12a system.

crRNA spacer sequences were designed within the *omp25* target region according to the PAM requirements of Cas12a. crRNA1 was designed using the online crRNA design platform provided by Shenzhen Yizhi Biotechnology (Shenzhen, China).^1^ The selected crRNA1 spacer had a platform-reported design efficiency score of 62 and a GC content of 52%. crRNA2 was independently designed using SnapGene based on the *omp25* target sequence and the presence of a Cas12a-compatible PAM; therefore, no platform-derived efficiency score was available for crRNA2. Both candidate crRNAs were synthesized by General Biosystems Co., Ltd. (Anhui, China) and experimentally evaluated in combination with the RPA products. Final primer-crRNA selection was based on observed amplification and fluorescence performance rather than in silico design parameters alone. Sequences are listed in Table 1.

**Table 1:** RPA primer and crRNA sequences.

Primer/crRNA	Sequence (5′–3′)	Amplicon length (bp)
RPA-F1	ATCGTCTCGGCTGCGCTGCTGCCGTTCTCTG	187
RPA-R1	GCTTGATGCTGCCAACGGTGCTGGTCTTGGC	
RPA-F2	CTACGACCTGAACCCGGTTAT	181
RPA-R2	GTACTGGGTGTAACGGTACTCAAC	
RPA-F3	GATCGTATACGGTGTTGAAGGTGATGCAGG	296
RPA-R3	TGTTGCCGTACTGGGTGTAACGGTACTCAA	
RPA-F4	GCCGACGCCATCCAGGAACAGCCTCCGGTTC	219
RPA-R4	ATCACCTTCAACACCGTATACGATCTGGTCC	
crRNA1	UAAUUUCUACUAAGUGUAGAUGUCGUCCAAGCCGUUGUUAAGCU	
crRNA2	UAAUUUCUACUAAGUGUAGAUAAGGCUCGCUGCGUGCCCGCGUC	

### 2.5 RPA amplification and primer verification

The *omp25* positive standard plasmid was used as the template to evaluate four RPA primer pairs (RPA-F1/R1, RPA-F2/R2, RPA-F3/R3, and RPA-F4/R4). According to the manufacturer’s instructions, one lyophilized RPA reaction pellet was reconstituted with 5 μL nucleic-acid template and 16 μL nuclease-free water. Forward and reverse primers (10 μM each; 1 μL each) and 1.25 μL Mg^2+^ solution (280 mM) were added to the tube lid. After inversion mixing and brief centrifugation, reactions were incubated at 42 °C for 20 min. Amplification products were purified and analyzed by 1% agarose gel electrophoresis. Primer performance was assessed on the basis of expected product size, band intensity, and nonspecific background.

### 2.6 Screening of RPA primer-crRNA combinations

RPA products were used as templates for CRISPR/Cas12a detection. The 20-μL Cas12a reaction contained 0.5 μL LbCas12a Nuclease (10 μM), 0.5 μL crRNA (10 μM), 0.5 μL HOLMES-Fluo ssDNA Reporter 1 (10 μM), 2 μL 10 × HOLMES Buffer for Cas12a, 5 μL RPA product, and nuclease-free water to 20 μL. Reactions were incubated at 37 °C for 20 min in a real-time fluorescence PCR instrument, with fluorescence recorded every 30 s in the FAM channel. Endpoint fluorescence was also assessed using a blue-light illuminator and gel imaging system. Eight primer-crRNA combinations were compared under identical CRISPR/Cas12a reaction conditions. RPA products used for the eight combinations were generated using the same plasmid template concentration (2.72 × 10^9 copies/μL) and the same RPA reaction conditions. Each combination was evaluated in three independent reaction replicates, with each replicate prepared and reacted separately. The final RPA3-crRNA1 combination was selected after endpoint fluorescence data had been collected and statistically compared using one-way ANOVA followed by Tukey’s multiple-comparison test, together with the visual fluorescence performance of the coupled assay.

### 2.7 Verification of the RPA-CRISPR/Cas12a detection system

To evaluate whether fluorescence generation required the key components of the RPA-CRISPR/Cas12a system, a complete reaction and component-deletion controls were established. Cas12a protein, crRNA, RPA amplification product, or ssDNA reporter was omitted in separate controls. Fluorescence generation was evaluated after incubation to confirm that the observed signal required the simultaneous presence of the target amplification product, crRNA, Cas12a protein, and ssDNA reporter.

### 2.8 Optimization of Cas12a protein and crRNA concentrations

After identifying the best-performing primer-crRNA combination, the concentrations of Cas12a and crRNA were optimized using a two-factor concentration matrix. Final concentrations of Cas12a and crRNA were independently evaluated at 0, 50, 100, 150, 200, and 250 nM, while the ssDNA fluorescent reporter was maintained at a final concentration of 200 nM. Reaction mixtures containing the different Cas12a-crRNA concentration combinations were prepared, followed by addition of the RPA product as the target template. Each Cas12a-crRNA concentration combination was evaluated using three independent reaction replicates (*n* = 3). Reactions were incubated at 37 °C for 20 min. Fluorescence was visually assessed under 470-nm blue-light illumination and recorded using a gel imaging system, while endpoint fluorescence values were simultaneously measured using a real-time fluorescence PCR instrument. The concentration combination that produced the highest endpoint fluorescence response together with a clear visual fluorescence signal was selected as the optimal working condition for subsequent experiments.

### 2.9 Optimization of ssDNA reporter concentration

After optimization of Cas12a and crRNA concentrations, the ssDNA fluorescent reporter concentration was evaluated at final concentrations of 0, 50, 100, 150, 200, and 250 nM while other reaction conditions were held constant. Reactions were performed at 37 °C for 20 min, and fluorescence was observed using a blue-light illuminator and recorded using a real-time fluorescence PCR instrument. Each concentration was evaluated in three independent reaction replicates (*n* = 3).

### 2.10 Experimental specificity analysis

The specificity of the optimized assay was evaluated using the recombinant *omp25* plasmid as the positive control and nuclease-free water as the negative control. Nucleic acids from infectious bovine rhinotracheitis virus (IBRV), *Chlamydia abortus*, *Salmonella Typhimurium*, *Listeria monocytogenes*, and *Campylobacter fetus subsp. venerealis* were included as non-target templates. These organisms were selected because they are associated with bovine reproductive disease and are relevant to differential diagnosis, and confirmed positive nucleic-acid materials for these pathogens were available in the laboratory during the study period. Phylogenetically related taxa were evaluated separately by in silico analysis of the *omp25* target region. Each condition was evaluated using three independent reaction replicates (*n* = 3), with each replicate prepared and reacted separately, and endpoint fluorescence values were analyzed using GraphPad Prism 9.5.1.

### 2.11 In silico specificity analysis of the *omp25* target region

To complement the experimental specificity assessment, the *omp25* target region was compared in silico with homologous sequences from selected phylogenetically related taxa. The 642-bp *omp25* region of *Brucella abortus* strain 2308 (NC_007618.1:706319–706,960) was used as the reference sequence. For the purposes of sequence-selectivity analysis, the intended target group comprised brucellosis-associated *Brucella* taxa, whereas species formerly classified within the genus *Ochrobactrum* and subsequently reassigned to *Brucella* were treated as closely related non-target taxa.

Homologous regions from *Brucella anthropi* strain PBO (CP064063.1), *Brucella pseudogrignonensis* strain ESL2 (CP091783.1), *Brucella pituitosa* isolate MPB25535 (OZ264092.1), and *Brucella intermedia* strain NCTC12171 (UGSH01000003.1) were aligned with the reference sequence using SnapGene. Sequence variation across the homologous region, RPA primer-binding sites, and crRNA recognition regions was examined. In addition, the selected *omp25* target region was queried against *Yersinia enterocolitica* O:9 sequences using NCBI nucleotide BLAST to assess potential sequence similarity. These analyses were used to assess sequence-level compatibility or divergence only and were not interpreted as experimental evidence of assay detection or non-reactivity for the taxa examined.

### 2.12 Analytical sensitivity

The positive plasmid (2.72 × 10^10 copies/μL) was serially diluted 10-fold from 2.72 × 10^10 to 2.72 × 10^0 copies/μL. Each dilution was tested with the optimized RPA-CRISPR/Cas12a assay using 5 μL of template per RPA reaction, with nuclease-free water as the negative control. Fluorescence was recorded after the reaction, and each concentration was evaluated in three independent reaction replicates (*n* = 3). Template input per reaction was calculated by multiplying the plasmid concentration by the 5-μL template volume. The lowest concentration at which all three independent reaction replicates produced visually detectable fluorescence and endpoint fluorescence values higher than the corresponding negative control was recorded as the lowest DNA concentration detected under the experimental conditions tested.

### 2.13 Preliminary agreement evaluation using bovine nasal swab surveillance samples

A total of 96 bovine nasal swab samples were obtained during routine official brucellosis surveillance conducted by the Ningxia Hui Autonomous Region Animal Disease Prevention and Control Center, China, from May 11 to May 15, 2026. The samples originated from routine surveillance activities conducted in Ningxia Hui Autonomous Region, China. Immediately after collection, each nasal swab was placed in phosphate-buffered saline (PBS) and stored at 4 °C until analysis. Laboratory testing was completed during the second week following sample collection. Sample inclusion was not stratified according to qPCR status, and qPCR results were not used as a criterion for sample selection. Bovine nasal swabs were used because they were the specimen type routinely collected through the surveillance program and were available for the present study. Nasal swab collection is minimally invasive and operationally convenient for herd-level sampling. The field-sample evaluation therefore focused on this surveillance matrix. Total nucleic acids were extracted using a viral DNA/RNA extraction kit (Xi’an Tianlong Science and Technology Co., Ltd., Xi’an, China) with an NP968-S automated nucleic acid extraction system (Xi’an Tianlong Science and Technology Co., Ltd., Xi’an, China), according to the manufacturer’s instructions.

All samples were tested in parallel using the RPA-CRISPR/Cas12a assay and a commercial *Brucella* Genus Real-time PCR Detection Kit (probe-based method; Qingdao Lijian Biotechnology Co., Ltd., Qingdao, China), which served as the comparator assay. The qPCR primers and probe were supplied as premixed reagents, and their sequences and exact target gene were not disclosed by the manufacturer. Each 25-μL qPCR reaction contained 20 μL reaction mixture and 5 μL extracted DNA or control. Amplification was performed at 37 °C for 2 min and 95 °C for 20 s, followed by 40 cycles of 95 °C for 10 s and 60 °C for 30 s, with FAM fluorescence collected at 60 °C. A run was considered valid when the positive control had a Ct value <35 and the negative control showed no Ct value. Samples with Ct < 37 and a characteristic amplification curve were considered positive, whereas samples with no Ct value were considered negative. Samples with 37 ≤ Ct ≤ 40 were further evaluated according to the qPCR interpretation criteria, including repeat testing and assessment of amplification-curve morphology. A sample was classified as positive when a reproducible Ct ≤ 40 was accompanied by a characteristic amplification curve consistent with true amplification. Late instrument-generated Ct values without a characteristic amplification curve were not considered positive.

For the RPA-CRISPR/Cas12a assay, visual fluorescence under blue-light illumination was used as the primary qualitative readout. Sample classification was based on visual fluorescence relative to the concurrent negative control under the same imaging conditions. A sample was classified as positive when a visually distinguishable yellow-green fluorescence signal was observed above the background fluorescence of the negative control. Faint background fluorescence that was not clearly distinguishable from the negative control was not considered positive. Endpoint fluorescence values were recorded as supportive measurements of signal intensity; however, no fixed RFU cutoff was predefined or validated for clinical sample classification. Agreement between the RPA-CRISPR/Cas12a assay and the qPCR comparator was assessed using positive percent agreement (PPA), negative percent agreement (NPA), overall percent agreement (OPA), and Cohen’s kappa coefficient.

The samples had been collected for routine official veterinary surveillance rather than specifically for this research. No animals were experimentally infected and no additional animal sampling or procedures were performed for the purposes of this study.

### 2.14 Statistical analysis

For fluorescence optimization, specificity, and analytical-sensitivity experiments, each condition was evaluated in three independent reaction replicates (*n* = 3), and endpoint fluorescence data are presented as mean ± standard deviation (SD). Statistical analyses were performed using GraphPad Prism 9.5.1. For single-factor experiments, differences among groups were analyzed using ordinary one-way analysis of variance (ANOVA). Tukey’s multiple-comparison test was used for comparisons among RPA primer-crRNA combinations, whereas Dunnett’s multiple-comparison test was used when experimental groups were compared with a designated control. The Cas12a-crRNA concentration optimization was performed using a concentration matrix, and the working condition was selected based on endpoint fluorescence intensity together with the visual fluorescence response. A *p* value <0.05 was considered statistically significant. For agreement analysis of bovine nasal swab samples, qPCR was treated as the comparator method. Positive percent agreement (PPA), negative percent agreement (NPA), and overall percent agreement (OPA) were calculated together with their 95% confidence intervals. Exact 95% confidence intervals for PPA, NPA, and OPA were calculated using the Clopper–Pearson method. Cohen’s kappa coefficient was calculated as an additional measure of agreement between the two assays.

## 3 Results

### 3.1 Conservation of the *omp25* target region

Multiple-sequence alignment of the 642-bp *omp25* target region showed high overall sequence conservation among the representative *Brucella* sequences examined (Figure 1). No nucleotide differences were observed between the *B. abortus* 2308 reference sequence and the corresponding regions of *B. abortus* 544, S19, A19, and RB51. Compared with the reference sequence, *B. melitensis* 16M contained four nucleotide mismatches, *B. suis* 1330 contained two mismatches, *B. canis* ATCC 23365 contained three mismatches, and *B. ceti* DDEO02 contained two mismatches. The *B. ovis* ATCC 25840 sequence aligned over 606 bp of the reference region and contained three nucleotide mismatches and one gap.

The observed variations were distributed at several positions across the 642-bp target region, and some overlapped candidate RPA primer-binding or crRNA recognition sites (Figure 1). Overall conservation supported further experimental evaluation of the target region, whereas the final primer-crRNA combination was determined by coupled assay performance rather than sequence conservation alone.

The 642-bp target region was fully conserved in the vaccine strains A19, S19, and RB51 relative to *B. abortus* 2308, supporting predicted sequence compatibility of these strains with the assay recognition region; however, experimental detection of these vaccine strains was not directly demonstrated in this study. Accordingly, the assay would not be expected to distinguish vaccine-derived from wild-type *Brucella* DNA on the basis of this target and is not intended for DIVA applications.

### 3.2 RPA primer amplification

All four RPA primer pairs amplified products of the expected sizes from the *omp25* standard plasmid. Expected amplicon lengths were 187, 181, 296, and 219 bp for RPA-F1/R1, RPA-F2/R2, RPA-F3/R3, and RPA-F4/R4, respectively (Figure 2). Because all four primer pairs showed detectable amplification, they were retained for subsequent screening with the candidate crRNAs.

**Figure 2:**
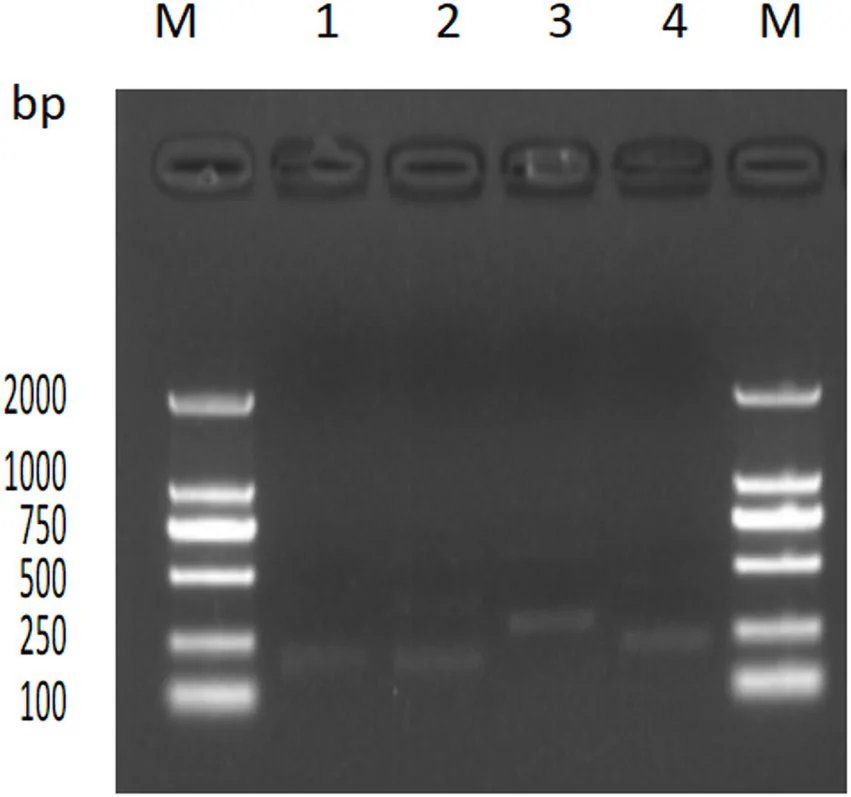
RPA amplification using four candidate primer pairs. M, DL2000 DNA marker; lanes 1–4, RPA-F1/R1, RPA-F2/R2, RPA-F3/R3, and RPA-F4/R4, respectively. The expected amplicon sizes were 187, 181, 296, and 219 bp, respectively.

### 3.3 Screening of RPA primer-crRNA combinations

The four RPA products were evaluated with the two candidate crRNAs under the same CRISPR/Cas12a reaction conditions. Each of the eight primer-crRNA combinations was evaluated using three independent reaction replicates (*n* = 3). Among the eight combinations tested, RPA2-crRNA1, RPA3-crRNA1, and RPA3-crRNA2 produced evident fluorescence responses. RPA3-crRNA1 produced the highest observed endpoint fluorescence response and was selected for subsequent assay optimization (Figure 3). Thus, final primer selection was based on performance in the coupled RPA-CRISPR/Cas12a system rather than agarose-gel band intensity alone.

**Figure 3:**
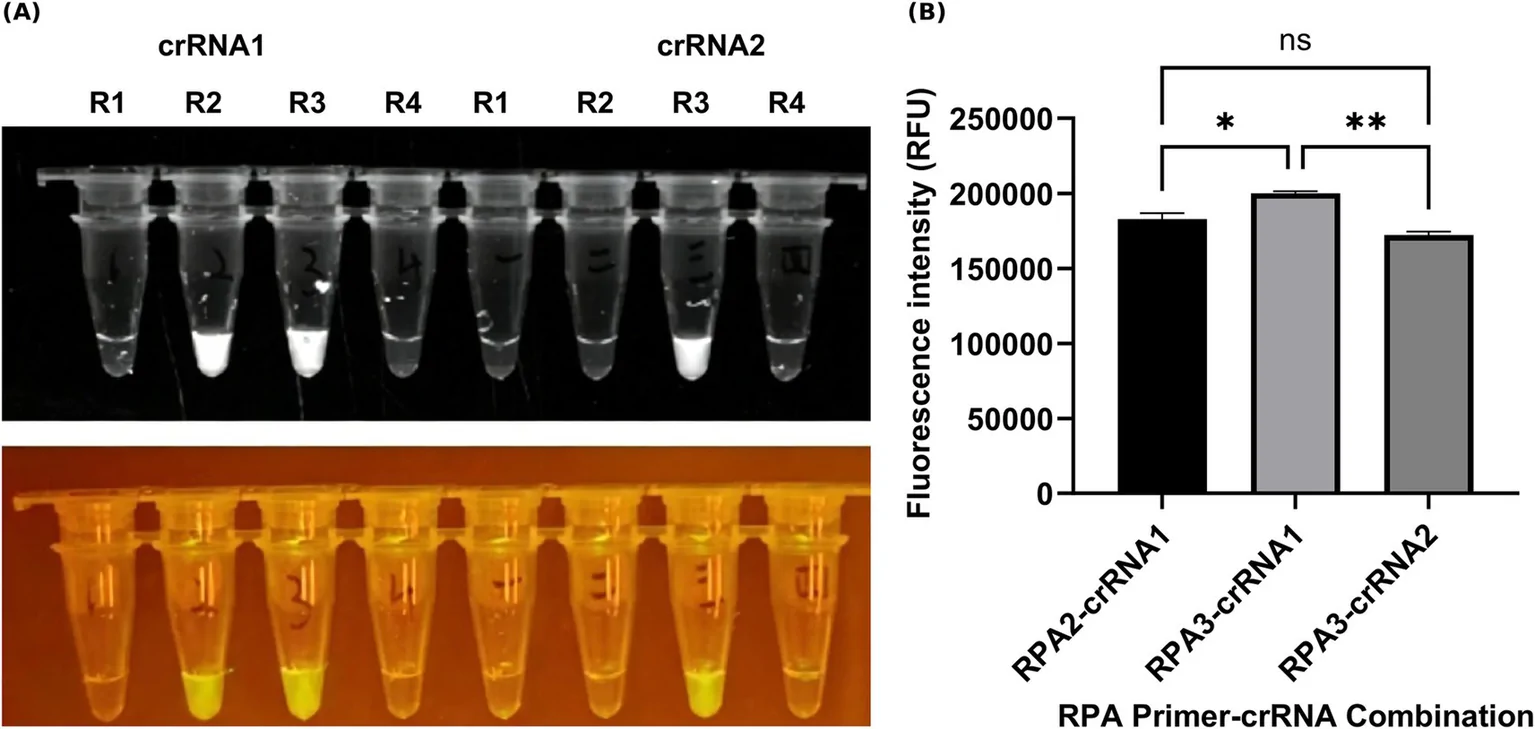
Screening of RPA primer-crRNA combinations in the CRISPR/Cas12a detection system. **(A)** Fluorescence images of the different RPA primer-crRNA combinations obtained using a gel imaging system and blue-light illumination. **(B)** Endpoint fluorescence intensities (RFU) measured after 20 min using a real-time fluorescence PCR instrument. Data are presented as mean ± SD from three independent reaction replicates (*n* = 3). Statistical comparisons were performed using one-way ANOVA followed by Tukey’s multiple-comparison test. *p < 0.05; **p < 0.01; ns, not significant.

### 3.4 Verification of the RPA-CRISPR/Cas12a detection system

Fluorescence output depended on the complete RPA-CRISPR/Cas12a system. A strong signal was detected only when Cas12a protein, crRNA, RPA amplification product, and ssDNA reporter were all present. No effective fluorescence signal was produced when any core component was omitted, indicating that the observed fluorescence under the tested conditions required the simultaneous presence of Cas12a, crRNA, RPA amplification product, and ssDNA reporter (Figure 4).

**Figure 4:**
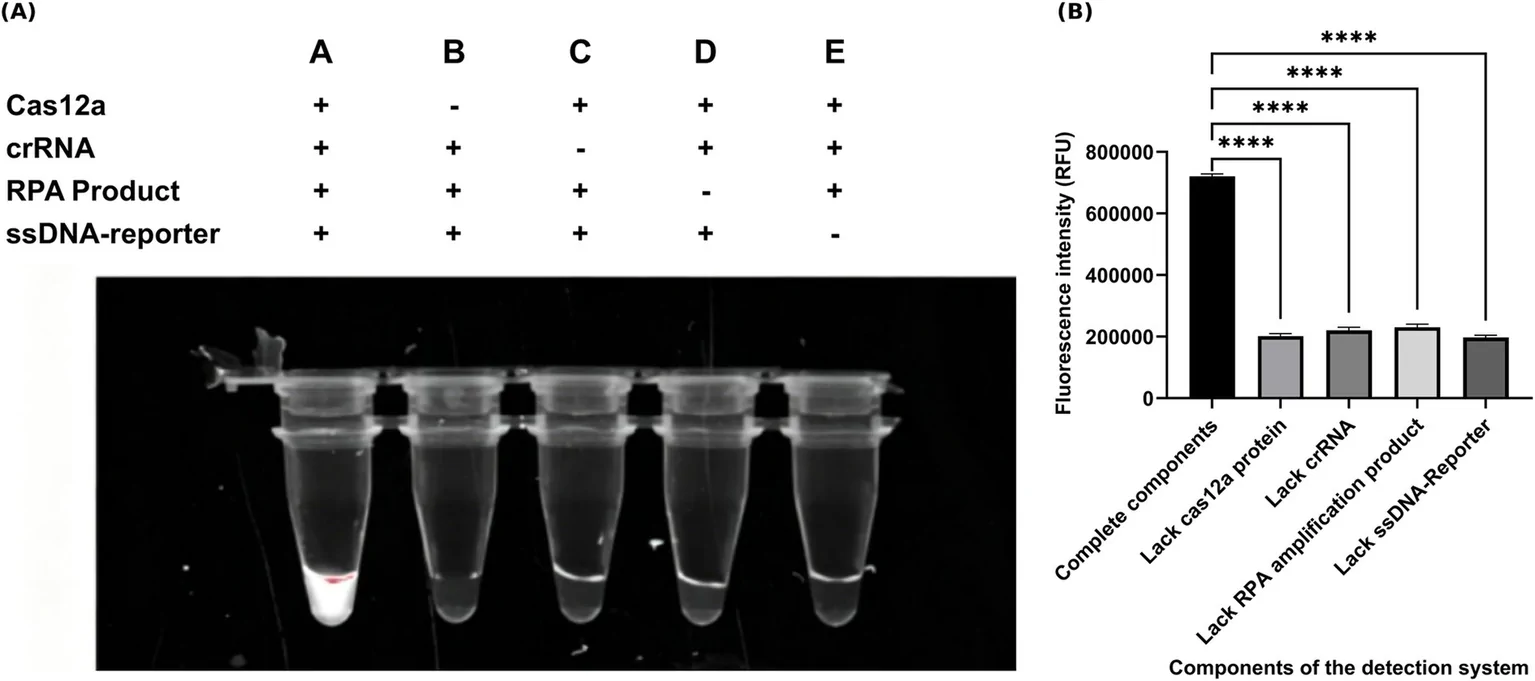
Verification of the RPA-CRISPR/Cas12a detection system. **(A)** Fluorescence images of the complete reaction and component-deletion controls lacking Cas12a, crRNA, RPA amplification product, or ssDNA reporter. **(B)** Endpoint fluorescence intensities (RFU) measured after 20 min. Data are presented as mean ± SD from three independent reaction replicates (*n* = 3). Statistical comparisons between the complete reaction and each component-deletion group were performed using one-way ANOVA followed by Dunnett’s multiple-comparison test. ****p < 0.0001.

### 3.5 Optimization of Cas12a protein and crRNA concentrations

Different Cas12a and crRNA concentrations affected endpoint fluorescence intensity. Within the tested concentration matrix, the highest fluorescence response was obtained at 250 nM Cas12a and 200 nM crRNA in the 20-μL reaction. This combination also produced a clear visual signal and was selected as the working concentration for subsequent experiments (Figure 5).

**Figure 5:**
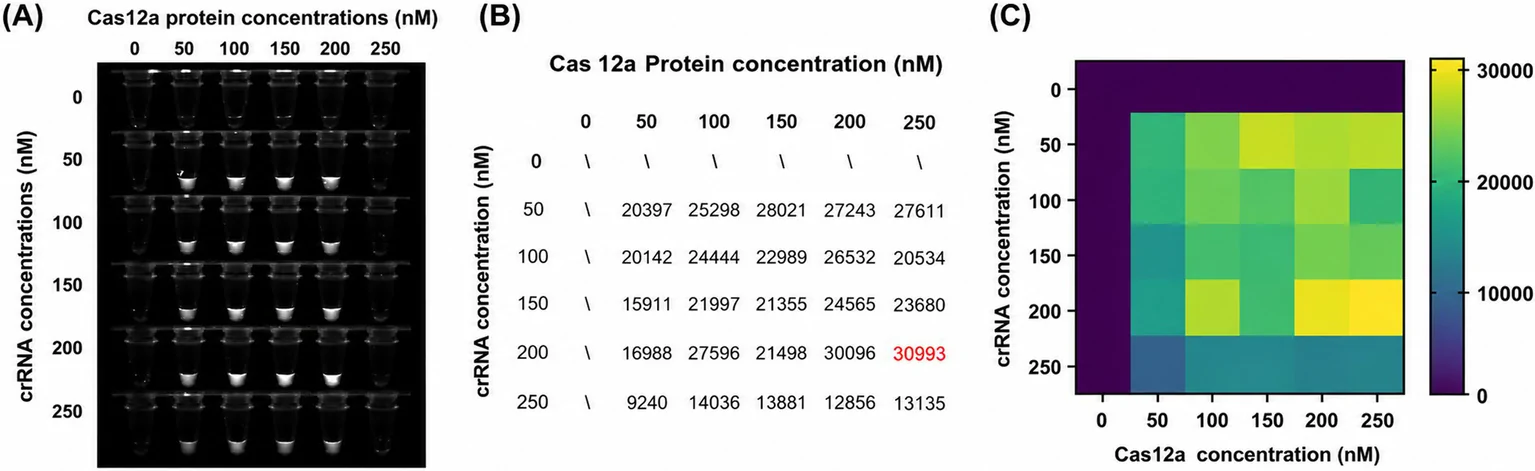
Optimization of Cas12a and crRNA concentrations. **(A)** Fluorescence images obtained with different Cas12a-crRNA concentration combinations. **(B)** Matrix of mean endpoint fluorescence intensities (RFU) for the tested concentration combinations. **(C)** Heatmap of the corresponding mean endpoint fluorescence intensities. Cas12a and crRNA were each evaluated at final concentrations of 0, 50, 100, 150, 200, and 250 nM. Each concentration combination was evaluated using three independent reaction replicates (*n* = 3). No inferential statistical comparison was performed for the two-factor concentration matrix. Within the tested concentration matrix, 250 nM Cas12a and 200 nM crRNA produced the highest endpoint fluorescence response together with a clear visual fluorescence signal and were selected as the working concentrations.

### 3.6 Optimization of ssDNA reporter concentration

Visible fluorescence was observed under blue light when the final reporter concentration was at least 100 nM. Endpoint fluorescence increased with increasing reporter concentration. Although 250 nM produced the highest endpoint signal, 200 nM generated a clear visual response and was selected as the working concentration after considering signal performance and reagent consumption (Figure 6).

**Figure 6:**
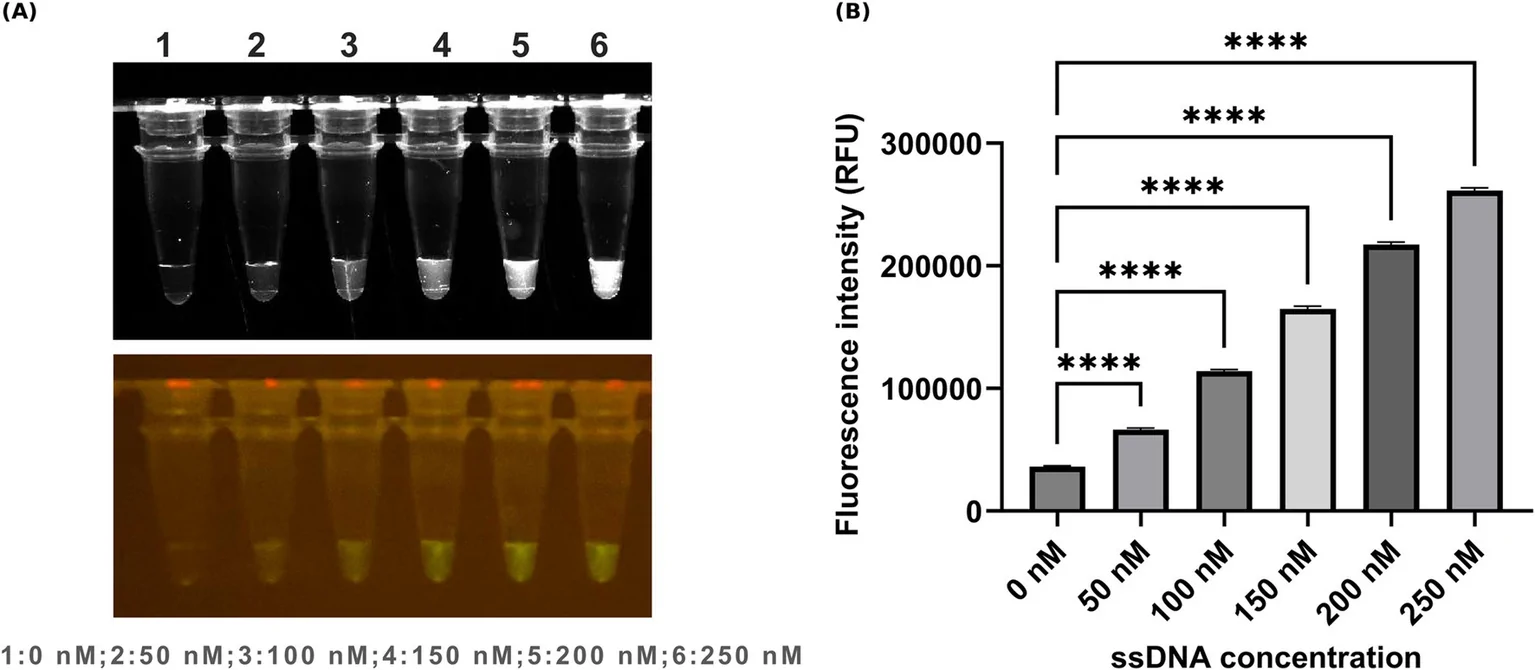
Optimization of ssDNA fluorescent reporter concentration. **(A)** Fluorescence images obtained at final ssDNA reporter concentrations of 0, 50, 100, 150, 200, and 250 nM under gel imaging and blue-light illumination. **(B)** Endpoint fluorescence intensities (RFU) measured after 20 min. Data are presented as mean ± SD from three independent reaction replicates (*n* = 3). Statistical comparisons with the 0 nM reporter group were performed using one-way ANOVA followed by Dunnett’s multiple-comparison test. ****p < 0.0001.

### 3.7 Experimental specificity

The optimized assay produced a clear yellow-green fluorescence signal in the *Brucella*-positive plasmid group. In contrast, IBRV, *Chlamydia abortus*, *Salmonella Typhimurium*, *Listeria monocytogenes*, *Campylobacter fetus subsp. venerealis*, and the negative control produced no detectable positive fluorescence signals. No cross-reactivity was observed with any of the five non-target organisms tested experimentally (Figure 7).

**Figure 7:**
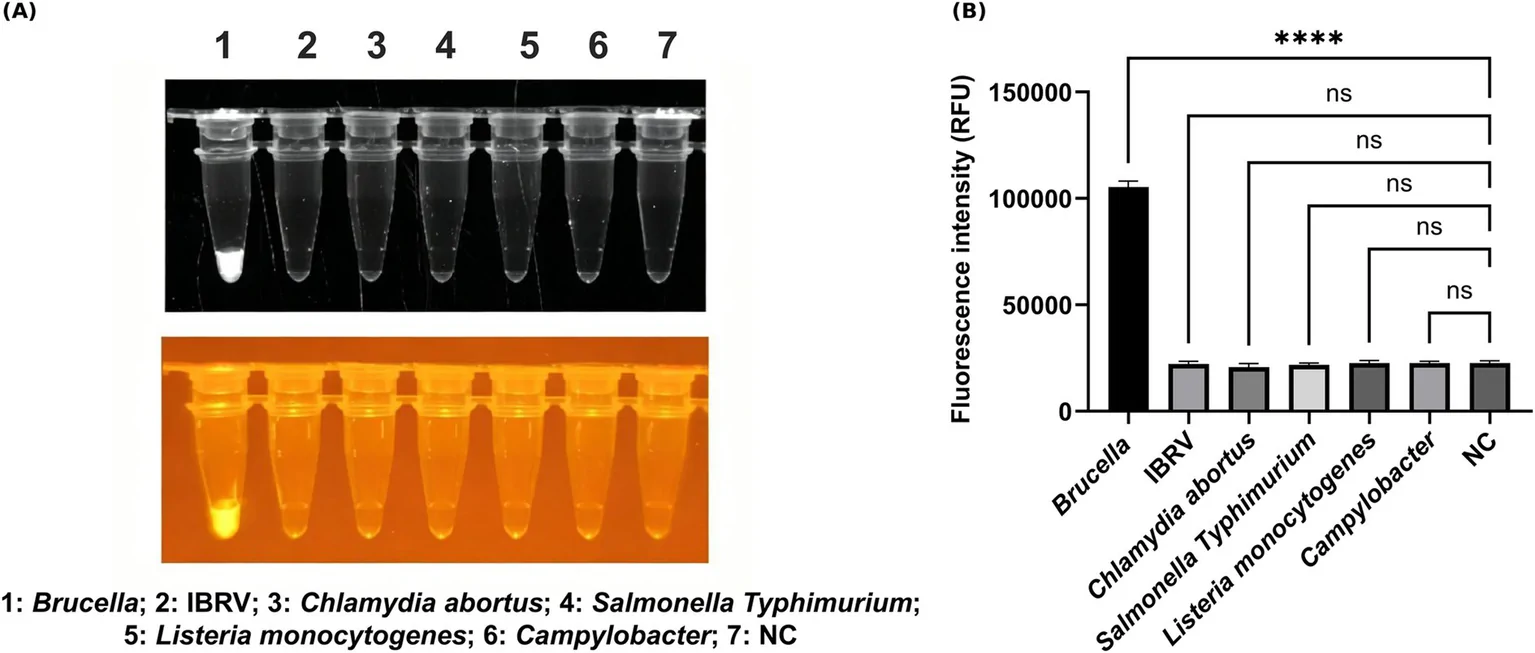
Experimental specificity of the RPA-CRISPR/Cas12a assay. **(A)** Fluorescence images of reactions containing the *Brucella* positive control, five non-target pathogen templates, and the negative control. Lane 1, *Brucella* positive plasmid control; lane 2, IBRV; lane 3, *Chlamydia abortus*; lane 4, *Salmonella Typhimurium*; lane 5, *Listeria monocytogenes*; lane 6, *Campylobacter fetus* subsp. *venerealis*; lane 7, negative control (NC). **(B)** Endpoint fluorescence intensities (RFU) measured after 20 min. Data are presented as mean ± SD from three independent reaction replicates (*n* = 3). Statistical comparisons with the negative control were performed using one-way ANOVA followed by Dunnett’s multiple-comparison test. ****p < 0.0001; ns, not significant.

### 3.8 In silico specificity of the *omp25* target region

Comparative sequence analysis showed substantial nucleotide variation between the *B. abortus* 2308 *omp25* reference region and homologous regions from the four selected phylogenetically related non-classical taxa (Figure 8). Across the aligned region, multiple mismatches and gaps were observed in all four taxa. Sequence differences were also present within the RPA primer-binding sites and crRNA recognition regions, including the RPA3 primer-binding sites and crRNA1 target region used in the final assay. In addition, no significant nucleotide similarity to the selected *omp25* target region was identified for *Y. enterocolitica* O:9 under the BLAST search conditions used. These results provide in silico support for a low predicted risk of sequence-based cross-reactivity with the related taxa examined. These findings are based on in silico sequence analysis and were not experimentally validated against these related taxa.

**Figure 8:**
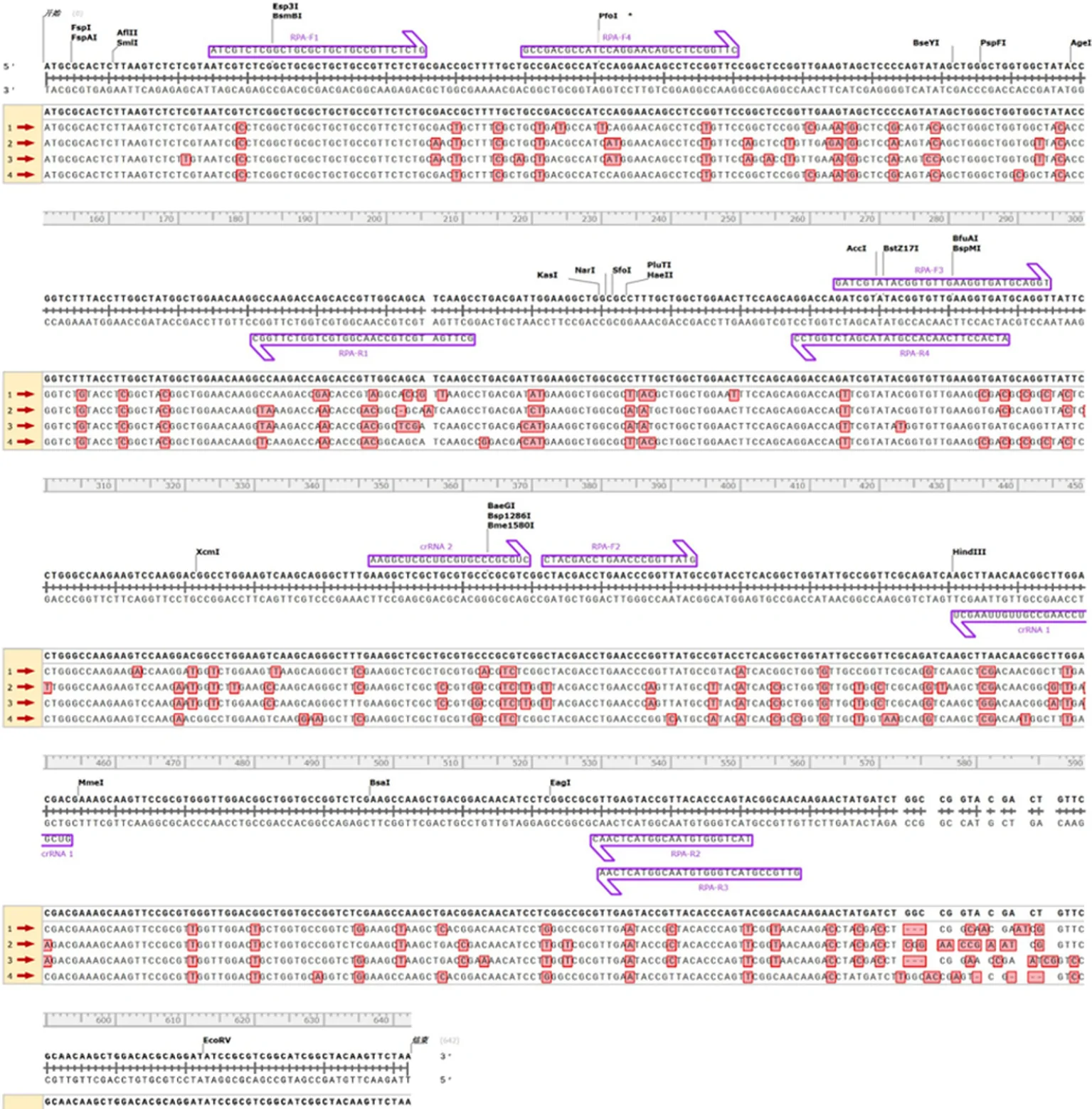
In silico comparison of the *omp25* target region with homologous sequences from selected phylogenetically related taxa. The 642-bp *omp25* region of *Brucella abortus* strain 2308 (NC_007618.1:706319–706,960) was aligned with homologous regions from *B. anthropi* strain PBO, *B. pseudogrignonensis* strain ESL2, *B. pituitosa* isolate MPB25535, and *B. intermedia* strain NCTC12171. Candidate RPA primer-binding sites and crRNA recognition regions are indicated, including the RPA3 primer-binding sites and crRNA1 recognition region used in the final assay. Nucleotide mismatches and gaps relative to the reference sequence are highlighted.

### 3.9 Analytical sensitivity

Endpoint fluorescence gradually decreased with decreasing plasmid concentration. Visible fluorescence remained detectable at an input plasmid concentration of 2.72 × 10^0 copies/μL, with an endpoint signal higher than that of the negative control. All three independent reaction replicates at this concentration met the positivity criterion used in this study, showing visible fluorescence together with an endpoint signal above the corresponding negative control. Because 5 μL of template was added to each RPA reaction, this concentration corresponded to an estimated template input of 13.6 copies per reaction. Therefore, the lowest DNA concentration detected under the experimental conditions tested was 2.72 copies/μL of input plasmid solution, equivalent to an estimated 13.6 copies per RPA reaction (Figure 9).

**Figure 9:**
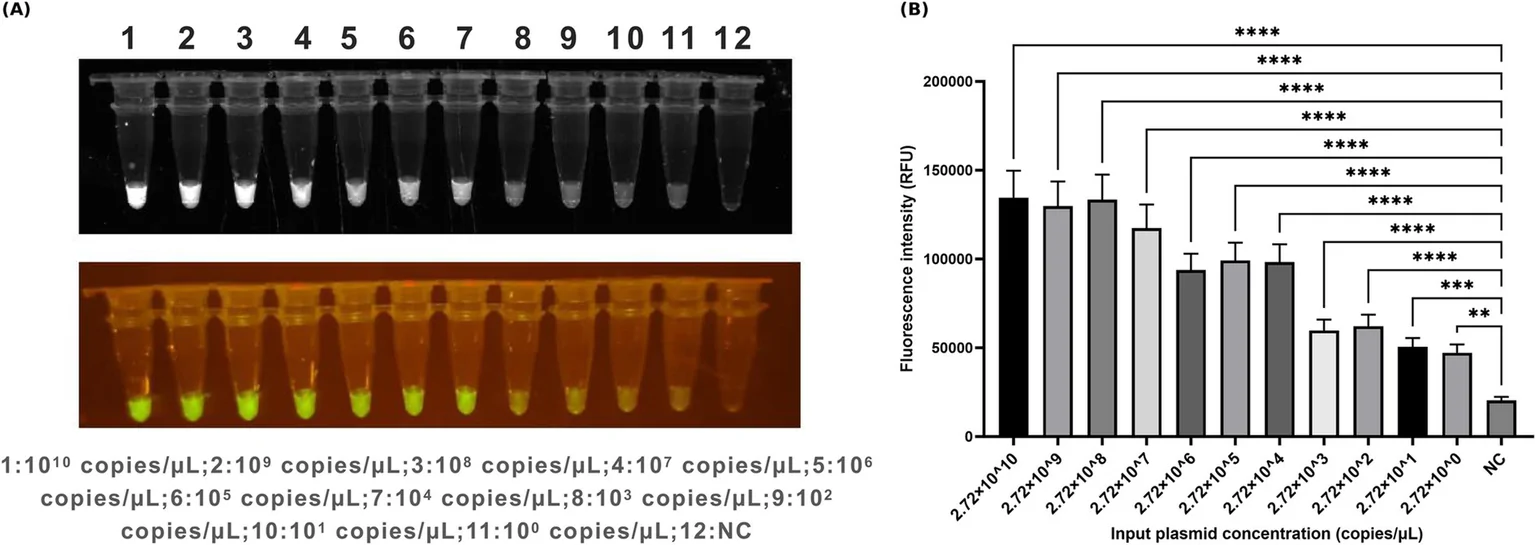
Analytical response of the RPA-CRISPR/Cas12a assay using serially diluted purified pUC19-*omp25* plasmid DNA. **(A)** Representative fluorescence images obtained using serial 10-fold dilutions of purified plasmid DNA from 2.72 × 10^10 to 2.72 × 10^0 copies/μL and a negative control (NC). **(B)** Endpoint fluorescence intensities (RFU) measured after 20 min using a real-time fluorescence PCR instrument. Because 5 μL of template was added to each RPA reaction, the corresponding template input ranged from 1.36 × 10^11 to 13.6 copies per reaction. Each concentration was evaluated using three independent reaction replicates (*n* = 3). A reaction was considered positive when visually distinguishable yellow-green fluorescence was observed relative to the negative control and the corresponding endpoint fluorescence value exceeded that of the negative control in the same run. Bars show mean ± SD. Statistical comparisons with the negative control were performed using one-way ANOVA followed by Dunnett’s multiple-comparison test. All three reaction replicates at 2.72 copies/μL met the positivity criterion. The lowest DNA concentration detected under the experimental conditions tested was 2.72 copies/μL, corresponding to an estimated 13.6 copies per RPA reaction. **p < 0.01; ***p < 0.001; ****p < 0.0001.

### 3.10 Agreement in bovine nasal swab surveillance samples

A total of 96 bovine nasal swab samples were tested in parallel using the RPA-CRISPR/Cas12a assay and the qPCR comparator. The qPCR comparator classified 48 samples as positive and 48 as negative. Among the 48 qPCR-positive samples, Ct values ranged from 33.21 to 38.99, with a median of 36.70 and an interquartile range of 35.28–37.69. Among the 48 qPCR-negative samples, 45 showed no Ct value, whereas three samples (Nos. 40, 65, and 79) generated late numerical Ct values of 39.86, 39.74, and 39.14, respectively, but lacked characteristic amplification curves and were therefore classified as negative.

The RPA-CRISPR/Cas12a assay classified 51 samples as positive and 45 as negative. All 48 qPCR-positive samples were also positive by the RPA-CRISPR/Cas12a assay. Three samples (Nos. 25, 81, and 82) were negative by qPCR but showed weak visually distinguishable fluorescence in the RPA-CRISPR/Cas12a assay and were therefore classified as discordant. Their endpoint fluorescence intensities were 158,777.03, 133,858.17, and 153,885.39 RFU, respectively. Endpoint fluorescence intensity varied among the clinical samples, and overlap was observed in the low-intensity range between samples with different qualitative classifications. Because no validated RFU cutoff was established for clinical sample classification, RFU values were used only as supportive measurements and were not used as an independent classification criterion. In the absence of an independent confirmatory method, the true status of the three discordant samples could not be resolved.

Agreement analysis yielded a positive percent agreement (PPA) of 100.00% (95% CI: 92.60–100.00%), a negative percent agreement (NPA) of 93.75% (95% CI: 82.80–98.69%), and an overall percent agreement (OPA) of 96.88% (95% CI: 91.14–99.35%). Cohen’s kappa coefficient was 0.938, indicating strong agreement between the two assays. These measures describe agreement with the qPCR comparator and should not be interpreted as definitive estimates of diagnostic sensitivity or specificity. The corresponding 2 × 2 agreement results are summarized in Table 2, and the RPA-CRISPR/Cas12a fluorescence results for all 96 samples are shown in Figure 10. Individual qPCR results and RPA-CRISPR/Cas12a endpoint fluorescence values are provided in Supplementary Table S1.

**Table 2:** Agreement between RPA-CRISPR/Cas12a and qPCR in 96 bovine nasal swab samples.

RPA-CRISPR/Cas12a result	qPCR positive	qPCR negative	Total
Positive	48	3	51
Negative	0	45	45
Total	48	48	96

**Figure 10:**
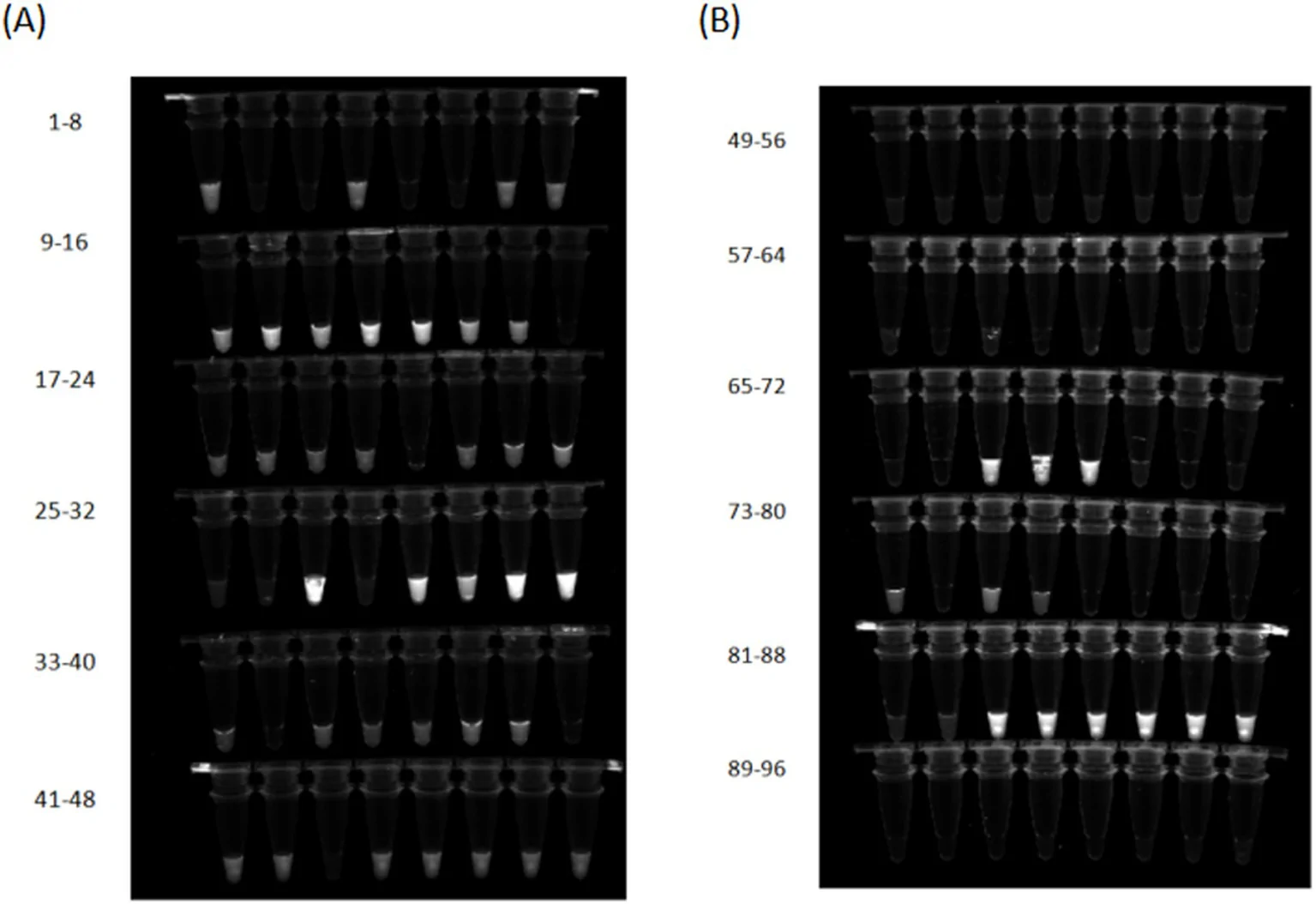
Fluorescence images of 96 bovine nasal swab samples tested using the RPA-CRISPR/Cas12a assay. **(A)** Samples 1–48; **(B)** Samples 49–96. Samples 25, 81, and 82 were negative according to the final qPCR classification but showed weak visually distinguishable RPA-CRISPR/Cas12a fluorescence and were classified as discordant relative to the qPCR comparator. Their endpoint fluorescence intensities were 158,777.03, 133,858.17, and 153,885.39 RFU, respectively. Clinical sample classification was based on visual fluorescence relative to the concurrent negative control under the same imaging conditions; endpoint RFU values were used as supportive measurements, and no validated RFU cutoff was applied.

## 4 Discussion

Early and rapid screening is an important component of brucellosis prevention and control in livestock production and public-health risk management. In this study, an *omp25*-targeted visual RPA-CRISPR/Cas12a assay was developed and evaluated. The amplification and CRISPR detection steps can be completed in approximately 40 min (20 min for RPA amplification and 20 min for Cas12a detection), and positive reactions can be visualized under 470-nm blue-light illumination. This reaction time excludes sample preparation, nucleic-acid extraction, and other pre-analytical procedures. Similar visual or dual-readout RPA-Cas12a systems have been explored for field-oriented pathogen detection (Jiang et al., 2025). The principal contribution of the present study is therefore not the introduction of RPA-Cas12a itself, but the evaluation of a conserved *omp25* region as an alternative target, experimental selection of a compatible primer-crRNA pair, and preliminary agreement testing in bovine nasal swab surveillance samples.

Selection of the molecular target and primer-crRNA combination strongly influences assay performance. RPA can generate nonspecific products under some reaction conditions (Lobato and O’Sullivan, 2018), making downstream sequence recognition valuable. All four primer pairs produced detectable RPA products, but only a subset of primer-crRNA combinations produced strong Cas12a fluorescence, with RPA3-crRNA1 performing best. This result supports screening primer and crRNA performance in the complete coupled workflow rather than selecting RPA primers solely from gel appearance. Comparative sequence analysis showed high overall conservation of the *omp25* target region among the representative classical *Brucella* sequences examined, although limited variation occurred at some candidate oligonucleotide-binding sites. The final crRNA1 recognition region was conserved among the classical *Brucella* sequences analyzed, whereas the final RPA3 primer sites showed limited variation in some strains. Experimental assay development, optimization, and analytical evaluation were performed using the recombinant *omp25* plasmid derived from *B. abortus* 2308. Thus, sequence conservation supports potential compatibility of the final RPA3-crRNA1 system with additional *Brucella* sequences, whereas actual detection of other *Brucella* species remains to be confirmed using representative genomic DNA or isolates. Furthermore, because *bcsp31*- and IS711-targeted RPA-CRISPR/Cas12a assays were not evaluated in parallel under identical experimental conditions, the present data do not establish that *omp25* provides superior analytical sensitivity, specificity, or overall assay performance. A standardized head-to-head comparison using identical template inputs, reaction conditions, and readout criteria would be required to determine whether *omp25* provides any practical performance advantage over these established targets.

Using purified plasmid DNA, the lowest DNA concentration detected under the experimental conditions tested was 2.72 copies/μL of input template, corresponding to an estimated 13.6 copies per RPA reaction. This value represents the lowest concentration detected under the purified-plasmid conditions used in this study and should not be interpreted as a formally validated limit of detection (LoD). A formal LoD determination would require repeated testing around the detection boundary using predefined statistical criteria. In addition, the plasmid-based experiment did not incorporate nucleic-acid extraction efficiency, potential DNA loss, matrix-associated inhibition, or other pre-analytical factors and therefore does not represent the sensitivity of the complete sample-to-result workflow. Previous *Brucella* RPA studies have reported low-copy analytical detection and multiplex differentiation of major *Brucella* species (Gumaa et al., 2019; Chang et al., 2023), while recent CRISPR-assisted *Brucella* assays have also achieved low-copy detection using fluorescence, visual, or lateral-flow readouts (Shen et al., 2025; Rasool et al., 2026; Deng et al., 2025; Dang et al., 2023). Because target sequence, template type, amplification chemistry, reporter format, and positivity criteria differ among studies, numerical detection levels should not be interpreted as directly equivalent. Accordingly, the value of 2.72 copies/μL should be interpreted specifically within the purified-plasmid experimental conditions evaluated here.

The Cas12a step provides sequence-dependent recognition after RPA amplification through PAM compatibility and crRNA-target complementarity (Paul and Montoya, 2020; Li et al., 2018; Swarts and Jinek, 2019; Mao et al., 2022). In the experimental specificity panel, the *Brucella*-positive template produced clear fluorescence, whereas IBRV, *Chlamydia abortus*, *Salmonella Typhimurium*, *Listeria monocytogenes*, and *Campylobacter fetus subsp. venerealis* remained negative. The in silico analysis further identified multiple differences in the selected related non-classical taxa, including at RPA3 and crRNA1 recognition sites. Because a positive signal requires both efficient RPA amplification and subsequent Cas12a activation, these sequence differences support a low predicted risk of cross-reactivity for the taxa examined. However, in silico mismatch patterns do not replace wet-laboratory specificity testing. A broader experimental panel that includes phylogenetically related taxa and diagnostically relevant organisms is still required.

The 96 bovine nasal swab samples were collected during routine regional surveillance. Nasal swabbing is rapid and minimally invasive and can be operationally convenient for herd-level sampling, but it is not an established replacement for the full range of specimens used in confirmatory diagnosis. Molecular detection of *Brucella* can vary by specimen type and testing strategy (Islam et al., 2025). Among the 96 bovine nasal swab samples, the RPA-CRISPR/Cas12a assay and the qPCR comparator produced concordant results for 93 samples, with a PPA of 100.00%, an NPA of 93.75%, an OPA of 96.88%, and a Cohen’s kappa coefficient of 0.938. These data represent a preliminary assessment of agreement in a single surveillance matrix rather than a comprehensive validation of diagnostic performance. Only one specimen type from a specific routine-surveillance context was evaluated. The three discordant qPCR-negative/RPA-CRISPR/Cas12a-positive samples could not be adjudicated using an independent reference method; therefore, it is not possible to determine whether they represent false-positive RPA-CRISPR/Cas12a signals, false-negative qPCR results, analytical variability in the weak-signal range, or another source of variability. Endpoint fluorescence values should also be interpreted cautiously. Although stronger fluorescence signals were generally observed among qPCR-positive samples, RFU values varied among individual samples and partially overlapped between qualitative groups in the low-intensity range. Accordingly, a reliable positivity cutoff based solely on endpoint RFU could not be derived from the present dataset, and clinical sample classification was based on visual fluorescence relative to the concurrent negative control rather than on RFU alone. This visual readout may be subject to observer-dependent variability for weak signals, and prospectively validated objective interpretation criteria will be required in future studies. For this reason, the study reports PPA, NPA, OPA, and kappa rather than diagnostic sensitivity and specificity against a definitive reference standard.

Several limitations should be considered. Specimen matrix can influence nucleic-acid detection because target DNA yield, extraction efficiency, inhibitor content, and matrix-associated background may differ among sample types. The present field evaluation included only bovine nasal swabs; therefore, assay performance in other specimen types used in brucellosis investigations, including blood, milk, vaginal swabs, and tissues, remains to be established. No inhibitor-spiking or cross-matrix comparison was performed in this study. The wet-laboratory specificity panel was limited, and several phylogenetically related taxa were evaluated only by in silico sequence analysis rather than experimental testing. The selected *omp25* target region was conserved in the analyzed sequences of vaccine strains A19, S19, and RB51; therefore, the assay would not be expected to distinguish vaccine-derived from wild-type *Brucella* DNA on the basis of this target and is not suitable for DIVA applications. Finally, the current assay employs a two-step open-tube workflow in which the RPA amplification product must be manually transferred to the subsequent Cas12a detection reaction. This post-amplification tube opening increases the risk of amplicon aerosol contamination and may compromise the practicality of the assay in decentralized or point-of-care settings. Closed-tube or one-pot RPA-CRISPR formats, in which amplification and Cas12a-mediated detection are integrated within a single reaction vessel without post-amplification product transfer, have been developed for other pathogens and may provide a useful strategy for reducing contamination risk and improving field applicability in future assay development (Gao et al., 2025; Liu et al., 2023; Zhao et al., 2023).

In summary, the *omp25*-based RPA-CRISPR/Cas12a assay provides a rapid visual workflow for preliminary screening of brucellosis-associated *Brucella* DNA and showed high agreement with qPCR in the bovine nasal swab surveillance samples evaluated. The current evidence supports analytical and preliminary surveillance-sample validation rather than broad diagnostic validation, species identification, or vaccine/wild-type differentiation. Larger evaluations using additional matrices, broader specificity panels, independently resolved discordant samples, and a closed-tube workflow are warranted before routine application.

## Data Availability

The original contributions presented in the study are included in the article/Supplementary material, further inquiries can be directed to the corresponding authors.

## Footnotes

1
www.ezassay.com

## Supplementary material

Supplementary Figure S1 Full-length multiple-sequence alignment of the 642-bp *omp25* target region among representative *Brucella* sequences. The *B. abortus* strain 2308 sequence (NC_007618.1:706319–706,960) was used as the reference. The alignment includes representative sequences of *B. abortus* 544, S19, A19, and RB51; *B. melitensis* 16M; *B. suis* 1330; *B. canis* ATCC 23365; *B. ceti* DDEO02; and *B. ovis* ATCC 25840. Candidate RPA primer-binding sites and crRNA recognition regions are indicated. Nucleotide mismatches relative to the reference sequence are highlighted, and gaps are shown as dashes.
